# Double-Blind Randomized Efficacy Field Trial of Alum Precipitated Autoclaved Leishmania major (Alum-ALM) Vaccine Mixed With BCG Plus Imiquimod Vs. Placebo Control Group

**Published:** 2015

**Authors:** Mohammad BARATI, Mehdi MOHEBALI, Mohammad Hossein ALIMOHAMMADIAN, Ali KHMESIPOUR, Hossein KESHAVARZ, Behnaz AKHOUNDI, Zabihollah ZAREI

**Affiliations:** 1 *Dept.of Medical Parasitology and Mycology, School of Public Health, Tehran University of Medical Sciences, Tehran, Iran*; 2 *Center for Research of Infectious Diseases, AJA University of Medical Sciences, Tehran, Iran*; 3 *Center for Research of Endemic Parasites of Iran (CREPI), Tehran University of Medical Sciences, Tehran, Iran*; 4 *Dept. of Immunology, Pasteur Institute of Iran, Tehran, Iran *; 5 *Center for Research and Training in Skin Diseases and Leprosy, Tehran University of Medical Sciences, Tehran, Iran*; 6 *Meshkin-Shahr Research station, School of Public Health, Tehran University of Medical Sciences, Iran*

**Keywords:** Alum, *Leishmania major*, Vaccine, Canine visceral leishmaniasis, BCG, Imiquimod, Iran

## Abstract

***Background:*** Canine visceral leishmaniasis (CVL) is not only an emerging veterinary concern but also a public health threat in endemic areas. The aim of this study was to assess the efficacy, immunogenicity and safety of two doses of aluminum hydroxide (alum) precipitated Leishmania major (Alum-ALM) mixed with BCG plus imiquimod against CVL.

**Methods:** A total of 560 ownership dogs were serologically tested and 234 healthy dogs with no clinical signs of CVL, no anti-Leishmania antibodies and negative leishmanin skin test were selected and double-blind randomly injected intradermally either with 0.1 ml Alum-ALM (200µg protein) mixed with BCG (2 × 10^6^ CFUs) plus imiquimod (121 dogs) or with 0.1 ml of normal saline (113 dogs).

**Results:** The follow-up examinations showed that there was no side effect associated with the vaccination except one case. Strong skin test conversion were seen in vaccinated group (30.3%) compared to the control group (6.6%) at 22-24 weeks after the booster injection (p<0.001). The seroconversion was 16.3% (18/110) in vaccinated group and 26.4% (28/106) in control group after two transmission cycles but the difference was not significant (P=0.095). The efficacy rate based on seroconversion was 40.4 %.

**Conclusion:** Two injections of Alum-ALM mixed with BCG and imiquimod is safe, although decreases the seroconversion rate of CVL, but the overall efficacy was low.

## Introduction

Zoonotic Visceral leishmaniasis (ZVL) is a public health problem in endemic areas (1-2) and is fatal in humans and dogs (3). 

Domestic and wild canines are the major reservoir hosts for *L.* *infantum* in Iran (4-5). The seroprevalence of CVL has been shown to vary between 10 to 37% in Iran (6-7) and recently 23.4% in Meshkin-Shahr district (8). 

Leishmaniasis treatment option is limited and resistant is emerging (9-10), and it is recommended not to use human medicine in dogs (11).

Elimination of infected dogs is not an easy task and its effect on control of human VL is controversial (12-13). The leishmaniasis control strategies are expensive, difficult and sometimes are not possible due to unacceptability by the owners (14-16). Although, an effective vaccine is the solution for leishmaniasis control but there is no vaccine available against any form of leishmaniasis (17). 

Dogs are the important reservoir hostsof ZVL, and as such, dogs’ vaccination is an essential step for the control of the disease (18). Efforts to develop first generation Leishmania vaccines have been reached to several phase 1 to 3 clinical trials. The safety and immunogenicity of autoclaved *L. major *(ALM) and Alum-ALM plus BCG was assessed (19-22). A meta-analysis study showed that the overal efficacy of first generation vacines was limited. One of the major reasons for the low efficacy was lack of proper adjuvant (23-24). Imiquimod is a Toll-like receptor (TLR7) agonists and an immunomodulator which when topically applied imiquimod showed potential vaccine adjuvant effect (25-27). Imiquimod induce a Th1 type of response and protection against *L. major* murine model of leishmaniasis (27). Imiquimod is applied topically for the treatment of CL in human (28-31). 

In the current study, for the first time imiquimod was used along with Alum-ALM +BCG as an adjuvant in ownership dogs. The efficacy and immunogenicity of Alum precipitated autoclaved *Leishmania major* (Alum-ALM) vaccine mixed with BCG plus imiquimod against CVL were evaluated in a double blind randomized study carried out in an endemic area of Meshkin-Shahr district north-west of Iran.

## Materials and Methods

***Study design***

**Fig. 1:**
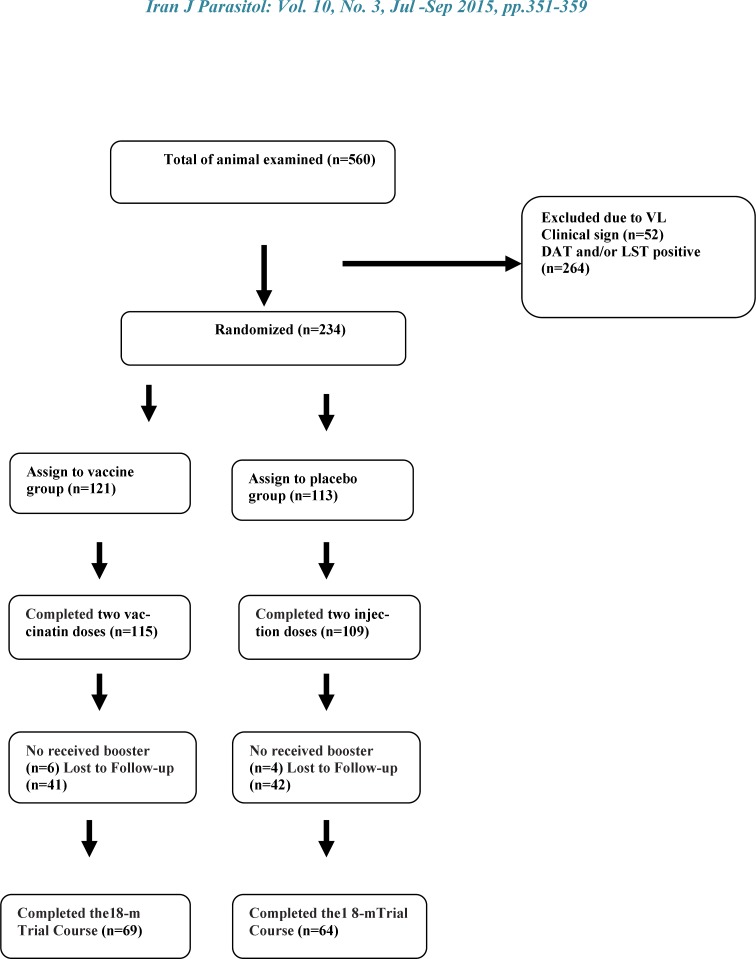
Trial profile

This is a double blind randomized; controlled field trial to evaluate efficacy, immunogenicity and safety of two doses of Alum precipitated autoclaved *L. major* (Alum-ALM) vaccine mixed with BCG along with imiquimod against CVL (Fig. 1).

***Site and Animal Selection***

The study was conducted from February 2011 to September 2013 in Meshkin-Shahr district in the north-west of Iran as an endemic area of VL. In this region, the transmission cycle period is July to September during sand fly activity (32-33). Sample size calculation was based on incidence rate about 18% (7) and vaccine efficacy was estimated to be 50%, confidence interval 95%, lost to follow up 20%, a total sample size was 234 animals. The potential candidate dogs were physically examined and each dog with signs or symptoms of VL was excluded. Blood sample was collected from each animal and used for direct agglutination test (DAT), the dog with any DAT titer was excluded. Afterward, the dog was with leishmanin skin tested and any dog with positive LST (≥1mm), was excluded. Dogs with DAT and LST negative results were selected and double blind randomly assigned to receive either 100 µl Alum-ALM (200µg) vaccine mixed with BCG (2 × 10^6^ CFUs) via intradermal injection or 100 µl normal saline. 

Initially, Imiquimod cream (5%) (Aldara, 3M, Canada) was applied topically 20 min before the vaccination.

For safety evaluation, the injected dog was examined physically and the vaccine injection site of the dog was checked.

***Experimental Vaccine***

Alum-ALM vaccine was produced at Razi Vaccine and Serum Research Institute, Hesarak, Iran under GMP guidelines. Briefly, promastigotes of *L. major* (MRHO/IR/75/ER) were cultured in RPMI1640 medium plus 20% FCS at 25 ºC. *Leishmania* promastigotes were harvested at stationary phase and were mixed with aluminum hydroxide (Alum) solution (1400 µg) in equal volume. The vials were autoclaved at 121 ºC for 15 min after aliquot.

At the time of vaccination, the content of vaccine vial (0.9 mL) was mixed well with 0.1 mL of freshly prepared BCG (Pasteur Institute of Iran, Tehran, Iran). The mixture was kept cool and used within 2 hours. The final injected dose of vaccine contained 200 µg of *Leishmania* protein and 1400 µg Alum, and 2 × 10^6^ CFUs of BCG. Before vaccine injection, 125 mg Imiquimod cream (5%) was applied topically in dogs groin and robbed in surface of 1-2 cm^2^ at the site of injection.

***Direct agglutination test (DAT)***

Blood sample was collected from each dog and DAT technique was used for the detection of anti *L. infantum* antibodies in sera according to the procedure described elsewhere (34-35). Briefly, for initial screening purposes, two-fold dilutions of sera were prepared from 1:80 to 1:320. Sera with titers 1:80 were diluted further to give maximum serum dilution of 1:20480. Wells with antigen only and no sera or known negative serum were used as negative control and known positive controls were used as positive control in each plate.

Anti-*L. infantum* antibody titers ≥1:320 were considered positive (6). DAT was used three times; for screening purposes to identify VL dogs and once after first transmission cycle and once after two transmission cycles to identify CVL.

***Leishmanin skin test***

TDR/WHO reference leishmanin manufactured at Pasteur Institute of Iran was used for skin testing according to the method previously described (36). The LST reaction was measured at 48-72 h after intradermal injection of 0.1ml in the right forearm, induration of with ≥5mm was considered as positive.

The LST was done two times; at screening, to detect exposure to the parasite, and at 22-24 weeks after the booster injection to evaluate in vivo vaccine immunogenicity.

***Parasitological confirmation***

Some of DAT positive dogs with different titers of anti-Leishmania antibodies were selected and after obtaining the informed consent of the owners, the dogs were sacrificed and parasitologically examined. Parasitology examination included microscopy and culture. For this purpose impression, smears were prepared from their spleen and liver. Prepared slides were fixed and stained usingGiemsa10% and the slides were checked for the presence of *Leishmania* amastigotes and also samples were cultured in NNN media and sub cultured in RPMI1640 supplemented with 10% foetal calf serum (FCS) with 100 IU /ml of penicillin and 100 µg/ml of streptomycin at 25 C for one month.

***Ethical approval***

The trial was reviewed and approved by the Ethics Committee of Tehran University of Medical Sciences (Ethic no.240/M/559) in accordance with Helsinki Declaration and guidelines.

***Data analysis***

Statistical analyses were performed using SPSS software (version 18) (Chicago, IL, USA). The Fisher’s exact test was used to evaluate between the groups and ^2 ^was used for analyses of differences between proportions. The difference of P-value was ≤0.05 was considered as significant.

Relative Risk (RR) is ratio of incidence rate in the vaccine group compared with the control (placebo) group. The vaccine efficacy was calculated with following formula: 100 × (1-RR).

## Results

Two hundred thirty four out of 560 ownership dogs with no anti-*Leishmania* antibodies and no response to leishmanin were selected and double blind randomly received either vaccine or placebo and 224 of the dogs received the booster injection at about one month later. 

After one transmission cycle, totally 8 dogs showed DAT positive, 5 dogs in vaccinated group and 3 dogs in placebo group. After the second transmission cycle, 46 dogs showed seroconversion using DAT. When the codes were broken, it was revealed that 18 dogs were from vaccinated group and 28 dogs were from placebo group. The seroconversion rate was calculated 16.3% (18/110) in vaccinated group and 26.4% (28/106) in control group using DAT technique. There was no statistically significant difference between two groups (P=0.095) (Table 1).The relative risk in the vaccine group was shown as 59.6%, and the efficacy of the vaccine calculated as 40.4 %.

The LST conversion rate in vaccine group converted to positive (30.3%) after the intervention (Table 2). A highly significant difference was observed in the LST between vaccinated and control groups (P=0.001). 

For safety assessment, the injection site was carefully examined and any Side effects were recorded after vaccination. The vaccine was generally safe and well tolerated except one case, which was observed an ulcer at the injection site. Mild reaction was observed locally in some vaccinated dogs that reactions were limited to the site of injection. 

A total of 25 seropositive dogs were used for autopsy which no growth *Leishmania* sp. in culture because of contamination but impression smear of 6 dogs (5 in vaccine group, 1 in placebo group) were positive.

No statistically significant difference was observed in the DAT positivity between vaccinated and control groups (P=0.095).

Statistically significant difference was indicated in the LST positivity between vaccinated and control groups (P=0.001). 

**Table 1:** Results of positive DAT after natural challenge of two transmission cycl*es*

***Group***	***No. examined (%)***	***DAT positivity (≥1:320)***
		***No. (%)***
*Vaccine*	*110 (50.9)*	*18 (16.3)*
*Placebo*	*106 (49.1)*	*28 (26.4)*
*Total*	*216 (100)*	*46 (21.3)*

**Table 2:** Results of LST (≥5 mm) at weeks 22-24 second dose vaccination

***Group***	***No. examined (%)***	***LST positivity (≥5)***
		***No. (%)***
*Vaccine*	*89 (54.3)*	*27 (30.3)*
*Placebo*	*75 (45.7)*	*5 (6.6)*
*Total*	*164 (100)*	*32 (19.5)*

## Discussion

Vaccine trials against leishmaniasis pioneered by South American scientists 75 years ago (37). Numerous *Leishmania* antigens from different laboratories from all over the word have been introduced as vaccine candidates. Experimental vaccines which were used against CVL includes first generation vaccines (killed *Leishmania* parasites and parasite proteins), second generation vaccines (recombinant *Leishmania* antigens) and third generation vaccines (DNA vaccines) (18, 38) but only a few of the vaccines reached to clinical trials (39-40).

Several first generation vaccines reached to phase 3 trials in humans and also were tested against canine ZVL (21--23, 41).

Successful development of an effective vaccine against leishmaniasis is mainly depends on selection of an appropriate adjuvant (23-24). In the current trial, imiquimod was used as an adjutant to enhance immune responses of Alum-ALM mixed with BCG against canine VL. To our knowledge through search on internet, there is no data available on the use of imiquimod on canine immune response.

In the present study, overall findings showed that Alum-ALM vaccine plus BCG and imiquimod was safe and well-tolerated .The incidence rate was calculated based on seroconversion. The seroconversion rate in the vaccine group was 16% and in control group was 26% after two transmission cycles. The efficacy rate was 40.4%.

Phase 1-2 study of Alum-ALM mixed with BCG was completed in human in Sudanese healthy individuals and showed to be safe and highly immunogenic in vivo and in vitro (42). PKDL rate is high in Sudan and believe to be a source of infection, treatment of PKDL is difficult so a phase 2 trial was designed in which Alum-ALM mixed with BCG was used to treat PKDL patients, the results of treatment with Alum-ALM plus BCG and chemotherapy was promising (43). 

The vaccine efficacy obtained in here was significantly less than our previous study in which the efficacy rate of a single injection of Alum-ALM plus BCG was much higher (69%). This difference may be due to various reasons such as the batch-to-batch variation of the vaccine. Other reason for discrepancy is that incidence rate was calculated based on seroconversion, which is not a true reflection of the disease, the real incidence could be reached by sacrificing the seroconverted dogs and search for *Leishmania* infection but due to ethical considerations and limited funds available, this part was not completed. In addition, another is the species of *Leishmania* parasite applied in vaccine, which in the current trial vaccine was based on *L. major* while ZVL caused by *L. infantumin* the endemic area of Meshkin-Shahr (6). Thus, it is possible that Alum-ALM vaccine will not be complete protective in these areas.

Application of topical imiquimod was done for the first time and no evaluation was performed to check if imiquimod was an immunomodulator and if so what type of Th1/Th2 response was initiated which is very crucial issue. Imiquimod might not act in dogs similar to murine model. When imiquimod was used to treat CL, the lesions are open but no data was generated to check the rate of imiquimod penetrate in the intact skin of dogs. Imiquimod was effective in treatment of New word CL but in Old world CL caused by *L. tropica*, imiquimod was used with systemic Glucantime® and showed not to increase low efficacy of Glucantime® which might be due to the fact that *L. tropica* lesions are mostly not ulcerated (25,31).

The route of injection also might be a factor to affect to outcome. In a study performed in murine model, application of topical imiquimod prior to subcutaneous injection with crude *Leishmania* antigen showed to enhance Th1 response and protection against *L. major* infection, while in the current study, vaccination was done intradermally (27).

In this study, immunization with two doses of the experimental vaccine induced a highly significant LST conversion rate, which was accordance with the previous study (21). 

Future we recommended studies on the predominant homologous species of *Leishmania* circulating in the endemic area and production of vaccine from these species and the use of effective and practical adjuvant in dogs.

## Conclusion

Vaccination with Alum-ALM plus BCG and imiquimod showed no significant side effect and was immunogenic when consider LST as an in vivo immune response reaction but efficacy rate was not as high as expected.
